# The factors influencing postoperative efficacy of anterior clinoidal meningioma treatment and an analysis of best-suited surgical strategies

**DOI:** 10.3389/fneur.2023.1097686

**Published:** 2023-03-16

**Authors:** Li-Hua Chen, Yong Xia, Fan Wei, Kai Sun, Hong-Zhi Huang, Ru-Xiang Xu

**Affiliations:** ^1^Department of Neurosurgery, Sichuan Provincial People's Hospital, University of Electronic Science and Technology of China, Chengdu, China; ^2^Department of Neurosurgery, Chinese Academy of Sciences Sichuan Translational Medicine Research Hospital, Chengdu, China

**Keywords:** anterior clinoidal meningiomas, tumor type, anterior clinoid process removal, optic canal opening, visual function

## Abstract

**Objective:**

To explore the influence of the type of anterior clinoidal meningioma on surgical strategy planning, surgical approach selection, and postoperative efficacy.

**Patients and methods:**

We conducted a retrospective analysis of the clinical data of 63 cases, including data on visual function, extent of tumor resection, and postoperative follow-up. Grade I and II approaches were selected according to the type of tumor. A univariate analysis of the factors influencing the extent of tumor resection, postoperative visual function, and postoperative relapse and complications was conducted.

**Results:**

Simpson Grade I–II total resection was seen in 48 cases (76.2%), with an overall relapse/progression rate of 12.7%. The tumor type and texture and the relationship between the tumors and adjacent structures were the main factors influencing total tumor resection (*P* < 0.01). The overall postoperative visual acuity improvement, stabilization rate, and deterioration rate were 76.2, 15.9, and 7.9%, respectively. Postoperative visual acuity level was significantly correlated with preoperative visual acuity level and tumor type (*P* < 0.01).

**Conclusions:**

Determining the type of tumor at a preoperative level and whether the optic canal and cavernous sinus are invaded can aid in the planning of detailed individualized surgical strategies.

## 1. Background

Anterior clinoidal meningiomas (ACMs) tend to extend along the anterior clinoid process (ACP) dura mater toward the optic canal and may be accompanied by hyperostosis of the ACP (1). Different types of ACMs have different growth patterns and can encase and invade the anterior circulation arteries, anterior visual pathway, or even the cavernous sinus (1–5). Early and satisfactory exposure of the optic nerve and internal carotid artery (ICA) can be difficult, causing an increased risk of intraoperative injury to the ICA and anterior visual pathway (6). The key to obtaining good exposure, increasing the extent of resection, and improving the prognosis lies in adequate preoperative evaluation, correct tumor typing, individualized surgical strategy development, and appropriate surgical approach selection. ACP removal and optic canal opening can lead to better visual effect and tumor resection rate to prevent tumor relapse (7). We retrospectively analyzed 63 ACM cases treated surgically from July 1999 to June 2021 and summarized and analyzed the factors affecting postoperative efficacy.

## 2. Patients and methods

Complete clinical and imaging data, intraoperative records, and follow-up data were available for all cases. This case group did not include recurrence, preoperative radiotherapy, and secondary ACM cases. All the family members signed their informed consent for the operation.

### 2.1. General information

In total, 63 patients with ACMs were included, and the group comprised of 15 males and 48 females aged 26–76.5 years, with an average age of 50.4 ± 13.2 years. The average disease course was 23.5 ± 19.3 (2–89) months. There were 27 cases with tumors on the left side, and 36 cases with tumors on the right side. The average preoperative Karnofsky Performance Score (KPS) was 89.3 ± 10.5 points.

### 2.2. Clinical indications

Visual acuity and visual field examinations were performed before and after surgery. Visual impairment and visual field disturbance were the most common first symptoms (61.9%, 39/63), as shown in Table 1.

**Table 1:** Clinical manifestations of 63 cases of ACMs and relationships with tumor types.

**Tumor type**	**Number of cases**	**Visual deterioration**	**Headache and dizziness**	**Visual field defect**	**Impaired memory**	**Papilledema**	**Eye movement disorders**	**Epilepsy**	**Hemiparesis**	**Psychiatric symptoms**
I	7	5	6	2	0	0	0	0	0	0
II	39	27	21	9	11	4	1	2	1	0
III	5	5	3	2	0	0	0	0	0	0
IV	12	12	11	10	4	7	5	2	3	2
Total	63	49	41	23	15	11	6	4	4	2

### 2.3. Preoperative imaging evaluation

**Figure 1:**
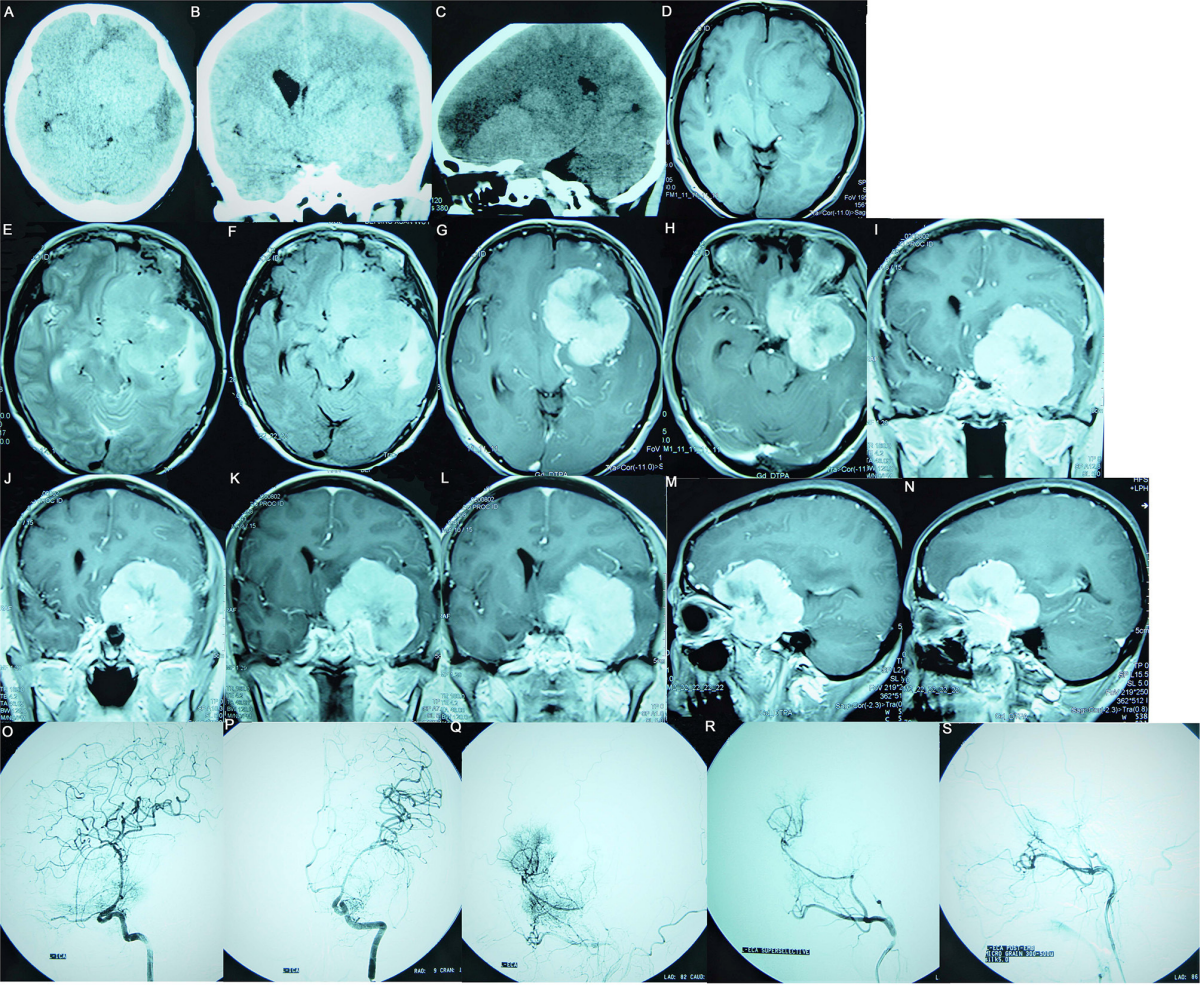
Preoperative images of a 61-year-old female patient, suggesting a meningioma whose base is rising from the ACP. **(A–C)** The CT plain scan images show a right parasellar isodense focus accompanied by hyperostosis of the ACP; the focus had a significant mass effect accompanied by left frontal and left temporal cerebral edema. **(D)** The T1WI image suggests isointensity; **(E)** The T2WI image suggests hyperintensity surrounded by a significant vascular flow void shadow. **(F)** The T2/FLAIR image suggests a hyperintense tumor surrounded by significant edema. **(G–N)** Enhanced MRI suggests that the tumor was significantly enhanced, with clear boundaries, uneven signal intensity, and calcification inside; the tumor encased the ICA and its branches and pushed the left posterior cerebral artery. **(G, H)** Enhanced axial images show that the tumor was centered around the ACP, growing toward the anterior and central skull base. **(I–L)** Enhanced coronal images show that the tumor pushed downward to invade the cavernous sinus, moved upward toward the Sylvian fissure, encased the ICA and its branches, and invaded the optic canal. **(M, N)** Enhanced sagittal images show that the tumor encased the ACP to grow forward and backward. **(O–S)** Preoperative DSA examination suggests that the tumor had a dual blood supply *via* the external and internal carotid arteries. **(O)** The preoperative internal carotid angiography shows the ICA siphon was compressed, the ACP and M1 segment had many newly formed blood vessels involved in the tumor's blood supply, and the ophthalmic artery was significantly thickened. **(P)** The entopic image shows that the M1 segment was elongated and thinned and moved upward. **(Q)** The external carotid arteriography shows tumor staining; **(R)** Super-selective arteriography of the external carotid artery shows that the middle meningeal artery was significantly thickened and involved in the tumor's blood supply. **(S)** The tumor staining disappeared after super-selective embolization of the external carotid artery.

Prior to surgery, all patients received enhanced magnetic resonance imaging (MRI) examinations and/or computed tomography (CT), computed tomography angiography (CTA), magnetic resonance angiography (MRA), and digital subtraction angiography (DSA) examinations (Table 2). Preoperative CT examinations of 53 cases revealed tumor calcification in 12 and reactive hyperostosis of the ACP in 31 cases. All 63 cases were examined by head MRI. Axial and coronal scans showed that the tumor base was located in different areas of the ACP (Figure 1). We divided edema-related cases into no or mild cerebral edema (edema thickness < 5 mm) and moderate to severe edema (edema thickness ≥5 mm). ACMs were categorized as soft or hard using the meningioma classification by Romani et al. (8). CTA, MRA, and DSA were performed in 27, 31, and 21 cases, respectively, before surgery. Referring to Mirimanoff et al. (9), the relationship between tumor and anterior circulation was divided into four levels: ① Level 0, where the tumor did not involve blood vessels, as observed in five cases; ② Level 1, where the tumor compressed and displaced arteries, with an intact arachnoid interface between the tumor and arteries, as observed in 12 cases; ③ Level 2, where the tumor encased or surrounded arteries (not stenotic), with an intact arachnoid interface between them, as observed in 39 cases; and ⑤ Level 3, where the tumor invaded arterial adventitia to cause stenosis, without an arachnoid interface between them, as observed in sevencases. With reference to the classification criteria for the relationship between tumors and arteries, the relationship between ACMs and the anterior visual pathway was also classified into four levels (Table 2). However, whether the tumor involves the optic nerve or optic canal cannot be fully and accurately assessed preoperatively. Therefore, it is necessary to detect whether the optic canal has tumor invasion or involves the optic nerve during the operation, so that corresponding treatment measures can be taken.

**Table 2:** Relationships between imaging features and extent of tumor resection in 63 cases of ACMs.

**Imaging feature**	**Number of cases (%)**	**Grade I–II total resection (%)**	**Grade III–IV resection (%)**	***X* ^2^**	***P*-value**
**Tumor type**					
I	7 (11.1)	1 (14.3)	6 (85.7)	24.935	<0.001
II	39 (61.9)	37 (94.9)	2 (5.1)		
III	5 (7.9)	4 (80)	1 (20)		
IV	12 (19.1)	6 (50)	6 (50)		
**Tumor size**					
Small	5 (7.9)	5 (100)	0	7.902	0.14
Medium	24 (38.1)	22 (91.7)	2 (8.3)		
Large	28 (44.5)	20 (71.4)	8 (28.6)		
Giant	6 (9.5)	1 (16.7)	5 (83.3)		
**Relationship with anterior circulation**					
0	5 (7.9)	5 (100)	0	23.049	<0.001
I	12 (19.1)	12 (100)	0		
II	39 (61.9)	31 (79.5)	8 (20.5)		
III	7 (11.1)	0	7 (100)		
**Tumor calcification**					
Yes	12 (19.0)	9 (75)	3 (25)	0.12	0.914
No	51 (81.0)	39 (76.5)	12 (23.5)		
**Hyperostosis of ACP**					
Yes	31 (49.2)	23 (74.2)	8 (25.8)	0.134	0.714
No	32 (50.8)	25 (78.1)	7 (21.9)		
**Involvement of cavernous sinus**					
Yes	9 (14.3)	1 (11.1)	8 (88.9)	24.515	<0.01
No	54 (85.7)	47 (87)	7 (13)		
**Involvement of optic canal**					
Yes	22 (34.9)	14 (63.6)	8 (36.4)	2.937	0.87
No	41 (65.1)	34 (82.9)	7 (17.1)		
**Classification of relationships between tumors and nerves**					
0	3	3	0	23.046	<0.01
I	14	14	0		
II	39	31	8		
III	7	0	7		
**Tumor texture**					
Soft	41	39	2	23.196	<0.01
Hard	22	9	13		
**Cerebral edema**					
None or mild	40 (63.5)	32 (80)	8 (20)	0.877	
Moderate to severe	23 (36.5)	16 (69.6)	7 (30.4)		
Total	63	48 (76.2)	15 (23.8)		

The maximum tumor diameter ranged from 1.5 to 7.4 cm, with an average of 4.2 ± 1.8 cm. According to the origin of ACM and the presence of cavernous sinus infiltration, ACM is divided into four types based on Al-Mefty's classification (2), and the criteria are as follows: ① Type I: ACMs arising from below the ACP, directly encasing and getting attached to the exposed adventitia of the ICA; ② Type II: ACMs arising from above or the lateral side of the ACP, with arachnoid separation from the ICA; ③ Type III: ACMs arising from the optic foramen area, growing toward the optic canal, and extending to the tip of the ACP, with arachnoid separation from the ICA; and ④ Type IV: ACMs invading and encasing both the entire ACP and parasellar area or invading the cavernous sinus.

### 2.4. Surgical methods and key points

#### 2.4.1. Selection of surgical approaches

**Figure 2:**
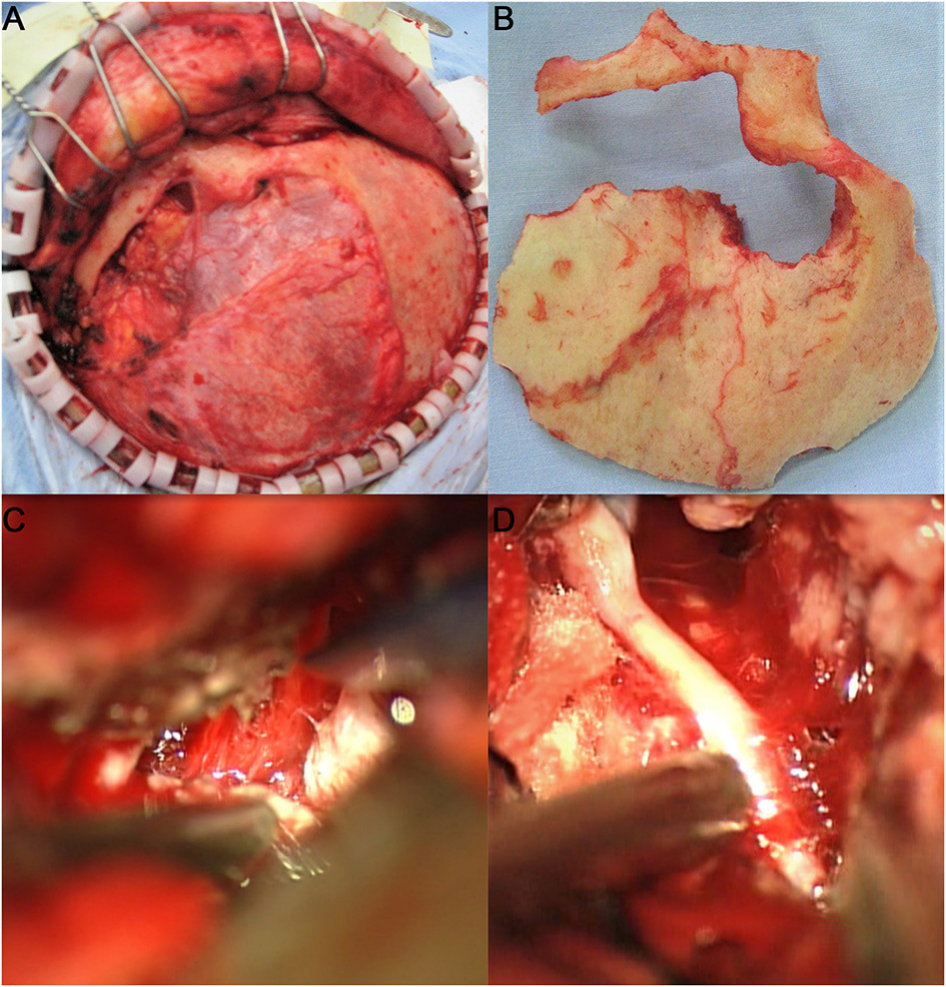
Adoption of the left frontotemporal–orbitozygomatic approach and intradural and epidural combination approach. One incisura was created on the trailing edge of the zygomatic process of the right frontal bone and two holes (outside the left orbit and on the left zygomatic arch) were drilled at the root of the left zygomatic arch to form a shape of ~8 × 10 cm with the bone flap. The anterior part of the bone flap was parallel to the anterior and central skull base. During surgery, the tumor base was found to originate from the left ACP and involve the lateral wall of the left cavernous sinus. The sphenoid ridge was first removed epidurally under the endoscope, and the medial side of the sphenoid ridge was removed to the extent possible. Then, the tumor was resected in blocks intradurally, while the tumor base was dissected. After the tumor was sufficiently decompressed, the ACP with hyperostosis was removed epidurally, and the optic canal was opened. The tumor was resected in sequence, in steps, and in blocks, intradurally. It was first separated from the anterior skull base and the left sphenoid ridge; then the first gap was opened, and the cerebrospinal fluid was released from the second gap on the right side to alleviate tension in the brain tissue. The tumor base was dissected from the left central skull base. It had invaded the superior petrosal sinus from the posterior and grown beyond the left petrosal ridge toward the left tentorium. It had also encased the middle cerebral artery and anterior cerebral artery. The tumor was then separated under the endoscope. It had squeezed posteriorly into the cerebral peduncle and compressed the ipsilateral cerebral peduncle to shift to the opposite side. It had completely invaded the cavernous sinus and the central skull base. It was resected under the endoscope in blocks. The dura mater of the central skull base was electro-coagulated, and the lateral wall of the left cavernous sinus was partially cut off to free part of the tumor in the cavernous sinus. The left oculomotor nerve and the trigeminal nerve's ophthalmic, maxillary, and mandibular branches were protected during surgery. The tumor in the cavernous sinus was also separated in steps and removed after electrocoagulation, and the bleeding in the cavernous sinus was stopped by gelatin sponge compression. **(A)** Adoption of the frontotemporal–orbitozygomatic approach; **(B)** orbitozygomatic bone flap; **(C)** intradural dissection of the ACP tumor base between the lateral side of the optic nerve and the internal carotid artery in the superior segment of the clinoid process; **(D)** the combination of antegrade and retrograde techniques used during surgery to dissect, separate, and protect the middle cerebral artery.

We divided the surgical approach into Grade I and Grade II (Table 3). The Grade I approach is concerned with reaching the tumor and choosing the right surgical approach according to the size and type of tumor. The Grade II approach is concerned with the selection of tumor resection methods, which are divided into the following three groups:

① Group A: A routine epidural resection of ACP and optic canal opening is performed, regardless of whether ACP is thickened or whether there is tumor infiltration in the optic canal. Among the group, there were totally 13 cases with only 8 cases of optic canal involvement. This is an improvement on the technology of Lee et al. (10).
② Group B: Thirty-six cases in this group underwent intradural tumor resection *via* the trans-Sylvian fissure approach. During the operation, six cases of ACP hyperosteogeny were intradural, 6 cases of tumor extended to the optic canal, and optic canal decompression was performed in the dura.
③ Group C: A total of 14 cases (giant or large ACM) underwent combined intradural and epidural approaches (Figure 2). Intradural decompression was performed first, and then ACP was removed and/or optic canal was opened.

**Table 3:** Selection and comparison of surgical approaches for 63 cases of ACMs.

**Item**	**Number of cases (%)**	**Level I approach (** ***n*** **, %)**			**X^2^**	***P*-value**	**Level II approach (** ***n*** **, %)**			***X* ^2^**	***P*-value**
		**Pterional**	**Frontal lateral**	**Orbitozygomatic**			**A**	**B**	**C**		
**Tumor type**											
I	7 (11.1)	5 (71.4)	2	0	22.969	<0.01	5	2	0	49.193	<0.01
II	39 (61.9)	18	17	4			3	32	4		
III	5 (7.9)	4 (80)	1	0			5	0	0		
IV	12 (19.1)	1	2	9 (75)			0	2	10		
**Tumor size**											
< 2	5 (7.9)	0	5	0	40.619	<0.01	4	1	0	32.050	<0.01
2–4	24 (38.1)	10	14	0			5	19	0		
4.1–6	28 (44.5)	18	3	7			4	16	8		
>6	6 (9.5)	0	0	6 (100)			0	0	6 (100)		
Total	63	28	22	13			13 (20.6)	36 (57.1)	14 (22.3)		

A: Different tumors need to choose different tumor resection methods: for ACMs with small tumors (diameter ≤ 4 cm), the anterior clinoid process and the opening of the optic canal should be routinely removed directly in the epidural. B: Most of the tumors were resected by simple intradural transsylvian approach. C: Only a few cases, six giant and eight large ACMs, required intradural decompression followed by epidural grinding of the anterior clinoid and opening of the optic canal.

#### 2.4.2. Main technical points

① ACP removal: If the ACP had hyperostosis, it had to be removed from the center and at the hyperostotic optic strut, followed by removal of the remaining ACP bone with a small, straight bone rongeur.
② Opticx canal opening: The roof and lateral wall of the optic canal were removed. After the optic nerve was completely exposed, the optic nerve sheaths were opened, and the falciform ligament was cut open to alleviate the optic nerve compression and to allow the optic nerve to stretch freely.
③ Tumor resection: The basilar cistern/Sylvian fissure cistern was opened as early as possible, and the tumor was internally decompressed. The tumor base and ACP dura mater base were treated first. Then, the boundaries between the tumor and the surrounding important nerves and blood vessels were separated along the arachnoid space, and the tumor was resected in sequence, steps, and blocks. When a tumor invaded the cavernous sinus, the tumor corridor could be followed into it; if a tumor was soft, it was resected to the extent possible.

#### 2.4.3. Postoperative follow-up

**Figure 3:**
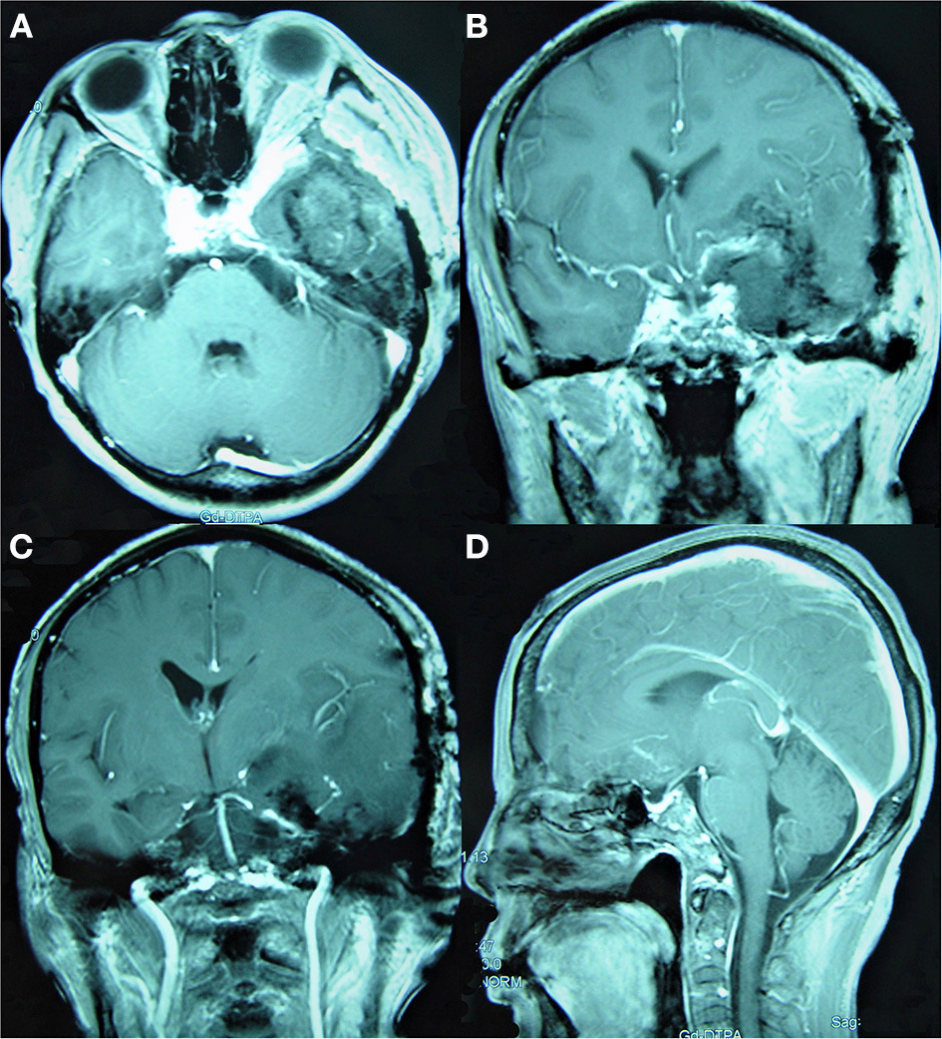
Simpson Grade III total resection of a tumor as suggested by enhanced MRI reexamination on the second day after surgery. **(A)** The enhanced axial image. **(B, C)** The enhanced coronal images show that the tumor invading the cavernous sinus was mostly resected and that the middle and posterior cerebral arteries were well protected. **(D)** The enhanced sagittal image shows that the tumor in the anterior and central skull base was satisfactorily resected.

**Figure 4:**
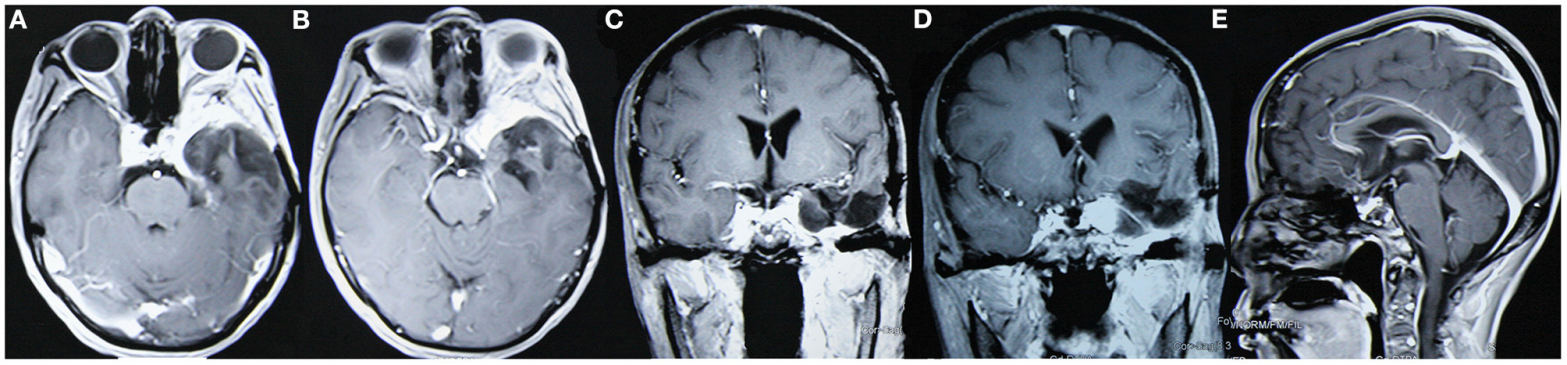
The tumor did not relapse as suggested in enhanced MRI reexamination 3 years after surgery. **(A, B)** Enhanced axial images show no tumor relapse in the central skull base; **(C, D)** enhanced coronal images show no tumor relapse in the cavernous sinus. **(E)** The enhanced sagittal image shows no tumor relapse in the anterior skull base.

The follow-up lasted for an average of 37.4 ± 13.8 (6–97) months. Enhanced MRI of the head was performed 3 days after surgery and 3 months after surgery (Figure 3), and MRI plain + enhanced scans were regularly taken every 6 months to 1 year thereafter, to assess any signs of local relapse (Figure 4). Three months after surgery, the visual acuity was assessed and categorized as “improved,” “unchanged,” or “deteriorated.” The Karnofsky Performance Score (KPS) was used to assess the patients' quality of life.

### 2.5. Statistical analysis

The SPSS Statistics 23.0 software was used for data analysis. Normally distributed measurement data are expressed as the mean ± standard deviation. Numerical data and ranked data were expressed by the case numbers and percentages, and the differences between groups were analyzed *via* univariate logistic analysis.

## 3. Results

### 3.1. Clinical and imaging features

The ACM commonly manifested as progressive visual impairment (77.8%, 49/63), usually occurring in one eye (71.4%, 45/63). There was a significant correlation of optic canal involvement with preoperative visual impairment, as all 22 cases with optic canal involvement had visual dysfunction before surgery. As shown in Table 2, the most common ACM was Type II (61.9%, 39/63). The ACMs often encased the ICA and/or middle cerebral artery (MCA) and anterior cerebral artery (ACA) complexes, accounting for 71.4% of cases (45/63); they invaded the anterior circulation blood vessels in only 11.1% of the cases (7/63; Level 3).

### 3.2. Tumor typing and surgical approach selection

Level I and II approaches were selected based on the tumor type and size, as shown in Table 3. The pterional approach or modified pterional approach was mostly selected for Type I and III ACM, the orbitozygomatic approach was selected for Type IV ACM, and the Level I approach was selected for giant ACMs. The ACM type and size closely determined the selection of Level I and II approaches, with significant differences (*P* < 0.01).

### 3.3. Analysis of factors influencing the extent of tumor resection

In 48 cases (76.2%), Simpson Grade I–II total resection was seen. The factors influencing the extent of tumor resection, cavernous sinus invasion, the tumor type, the relationship between the tumors and blood vessels/nerves, and the tumor's texture were the primary influential factors (*P* < 0.01; see Table 2). Furthermore, the extent of tumor resection was correlated with ACP removal and optic canal was opened (*P* < 0.05) but it had no significant relationship with the selection of Level II surgical approaches (A, B, or C; *P* = 0.963), as shown in Table 4.

**Table 4:** Relationship between intraoperative treatment and extent of tumor resection (case).

**Influencing factor**	**Number of cases (%)**	**Grade I–II total resection (%)**	**Grade III–IV resection (%)**	***X* ^2^**	***P*-value**
**Optic canal**					
Opened	22 (34.9)	20 (90.9)	2 (9.1)	4.037	0.045
Not opened	41 (65.1)	28 (68.3)	13 (31.7)		
**ACP removal**					
Yes	33 (52.4)	29 (87.9)	4 (12.1)	5.219	0.022
No	30 (47.6)	19 (63.3)	11 (36.7)		
**Level II approach**					
A	13 (20.6)	10 (76.9)	3 (23.1)	0.076	0.963
B	36 (57.2)	27 (75)	9 (25)		
C	14 (22.2)	11 (78.6)	3 (21.4)		
Total	63	48 (76.2)	15 (23.8)		

### 3.4. Analysis of factors influencing postoperative visual function

The overall postoperative visual acuity improvement, stabilization rate, and deterioration rate were 76.2% (48/63), 15.9% (10/63), and 7.9% (5/63), respectively, as shown in Table 5. Postoperative visual acuity level had a significant correlation with the preoperative visual acuity level and tumor type (*P* < 0.01) and had a specific correlation with optic canal opening during surgery (*P* = 0.029). In 22 cases with optic canal opening, 21 had visual acuity improvement (95.5%, 21/22). In the 41 cases without optic canal opening, 27 showed visual function improvement (65.9%, 27/41), and five showed deterioration (12.2%, 5/41) after surgery, as shown in Table 5.

**Table 5:** Analysis of factors influencing postoperative visual function (cases).

**Influencing factor**	**Number of cases (%)**	**Visual function improvement**	**Stabilization**	**Deterioration**	***X* ^2^**	***P*-value**
**Preoperative visual acuity level**						
< 0.1	7	1	3	3	29.093	<0.01
0.1–0.9	51	46	3	2		
≥1.0	5	1	4	0		
**Tumor type**						
I	7 (11.1)	3	4	0	23.380	<0.01
II	39 (61.9)	35	3	1		
III	5 (7.9)	1	3	1		
IV	12 (19.1)	9	0	3		
**Optic canal**						
Opened	22 (34.9)	21	1	0	7.062	0.029
Not opened	41 (65.1)	27	9	5		
**ACP removal**						
Yes	33 (52.4)	26	5	1	2.118	0.347
No	30 (47.6)	22	5	4		
**Level II approach**						
A	13	10	2	1	1.684	0.794
B	36	29	5	2		
C	14	9	3	2		
Total	63	48 (76.2)	10 (15.9)	5 (7.9)		

### 3.5. Analysis of factors influencing postoperative quality of life

The average KPS 6 months after surgery, at discharge, and before surgery was 91.3 ± 9.6 (60–100), 87.6 ± 7.8 (50–100), and 89.3 ± 10.5 (60–100) points, respectively. Six months after surgery, the KPS of 90.5% of the patients reached 80 points or more, and that of the remaining 9.5% reached 60–70 points. The tumor type was closely correlated with postoperative KPS (*P* < 0.01), while the selection of Level II approaches and the extent of tumor resection were not, as shown in Table 6.

**Table 6:** Analysis of factors influencing quality of life score (KPS; case).

**Influencing factor**	**Number of cases (%)**	**≥80 (%)**	** ≤ 70 (%)**	***X* ^2^**	***P*-value**
**Tumor type**					
I	7 (11.1)	5 (71.4)	2 (28.6)	13.850	0.001
II	39 (61.9)	39 (100)	0		
III	5 (7.9)	5 (100)	0		
IV	12 (19.1)	8 (66.7)	4 (33.3)		
**Level II approach**					
A	13	12 (92.3)	1 (7.7)	2.836	0.241
B	36	34 (94.4)	2 (5.6)		
C	14	11 (78.6)	3 (21.4)		
**Extent of tumor resection**					
Simpson I + II	48	44	4	0.332	0.622
Simpson III + IV	15	13	2		
Total	63	57 (90.5)	6 (9.5)		

A: Different tumors need to choose different tumor resection methods: for ACMs with small tumors (diameter ≤ 4 cm), the anterior clinoid process and the opening of the optic canal should be routinely removed directly in the epidural. B: Most of the tumors were resected by simple intradural transsylvian approach. C: Only a few cases, six giant and eight large ACMs, required intradural decompression followed by epidural grinding of the anterior clinoid and opening of the optic canal.

### 3.6. Factors influencing postoperative tumor relapse

The overall relapse/progression rate during the follow-up period was 12.7%. Among the 48 cases with Simpson Grade I–II total resection, only 1 experienced relapse during the follow-up period (1/48, 2.1%). The extent of tumor resection and tumor type had a significant correlation with postoperative relapse rate (*P* < 0.01), while the selection of Level II approaches did not (*P* = 0.346), as shown in Table 7.

**Table 7:** Analysis of factors influencing postoperative relapse (case).

**Influencing factor**	**Number of cases**	**No relapse**	**Relapse/progression**	***X* ^2^**	***P*-value**
**Tumor type**					
I	7	6	1	11.892	0.003
II	39	38	1		
III	5	4	1		
IV	12	7	5		
**Level II approach**					
A	13	13		2.247	0.346
B	36	30	6		
C	14	12	2		
**Extent of tumor resection**					
Simpson I + II	48	47 (97.9)	1 (2.1)	20.491	<0.01
Simpson III + IV	15	8 (53.3)	7 (46.7)		
Total	63	55 (87.3)	8 (12.7)		

A: Different tumors need to choose different tumor resection methods: for ACMs with small tumors (diameter ≤ 4 cm), the anterior clinoid process and the opening of the optic canal should be routinely removed directly in the epidural. B: Most of the tumors were resected by simple intradural transsylvian approach. C: Only a few cases, six giant and eight large ACMs, required intradural decompression followed by epidural grinding of the anterior clinoid and opening of the optic canal.

### 3.7. Analysis of factors influencing postoperative complications

Among the 63 ACM cases, 34 did not develop any complications after surgery, accounting for 54%; 25 had transient complications after surgery, all of which were cured by symptomatic treatment; and 4 had permanent dysfunction (without recovery 6 months after surgery), accounting for 6.3%. The tumor type, the relationship between the tumors and blood vessels/nerves, cavernous sinus involvement, and the choice of Level II approaches were the primary factors that influenced postoperative complications (*P* < 0.01), as shown in Table 8.

**Table 8:** Analysis of factors influencing postoperative complications.

**Influencing factor**	**Number of cases**	**No complication (%)**	**Transient complication (%)**	**Permanent complication (%)**	***X* ^2^**	***P*-value**
**Tumor type**						
I	7	1	5	1	30.918	<0.01
II	39	31	8	0		
III	5	1	3	1		
IV	12	1	9	2		
**Tumor size**						
Small	5	4	1	0	14.495	0.08
Medium	24	17	6	1		
Large	28	13	14	1		
Giant	6	0	4	2		
**Classification of relationships between tumors and blood vessels**						
0	2	2	0	0	32.444	<0.01
I	19	18	1	0		
II	36	14	21	1		
III	6	0	3	3		
**Classification of relationships between tumors and nerves**						
0	3	3	0	0	22.178	<0.01
I	14	13	1	0		
II	39	18	19	2		
III	7	0	5	2		
**Involvement of cavernous sinus**						
Yes	9	0	6	3	17.369	<0.01
No	54	34	19	1		
**Cerebral edema**						
None or mild	40	24	14	2	1.837	0.449
Moderate to severe	23	10	11	2		
**Level II approach**						
A	13	3	8	1	21.811	<0.01
B	36	28	8	0		
C	14	3	9	3		
Total	63	34 (54%)	25 (39.7%)	4 (6.3%)		

The classification of relationships between tumors and nerves/blood vessels is subject to the highest level of all.

## 4. Discussion

### 4.1. Influence of tumor typing on ACM

#### 4.1.1. Tumor type and surgical difficulty

Correct typing of ACMs aids in all surgical matters such as deciding which approach to take, difficulty prediction, risk estimation, and prognosis prediction (1). Sekhar (10) stated that something to be considered is whether the tumor invades the cavernous sinus and encases blood vessels. Risi et al. (11) proposed that tumor typing should take into consideration the direction of tumor extension. Whether the tumor involves the cavernous sinus and encases the ICA is an important factor influencing postoperative efficacy and, thus, should reflect in the typing. Nakamura et al. (12) divided ACMs into two groups, but this typing is too simplistic to accurately reflect the surgical difficulty and predict ACM prognosis. Xu et al. (3) divided ACMs into four types, with Type II ACMs being further divided into two subtypes, which becomes too complex. Based on Al-Mefty's classification, in this study, giant ACMs that invaded the cavernous sinus and had a tumor base covering the entire ACP were classified as Type IV. This typing can facilitate accurate image assessment and aid in preoperative development of surgical plans.

Type I ACMs encase the ICA and are directly attached to the adventitia of the ICA, without a clear arachnoid separation interface from blood vessels; separating a tumor directly adjacent to the adventitia of blood vessels can cause severe vasospasm and even directly injure the artery (6). When it comes to Type II and most Type III ACMs, they maintain the arachnoid layer between the tumor and blood vessels, providing a good interface for separating the tumor (6); it is less difficult to operate on such ACMs. The relationship between a tumor and the surrounding blood vessels is another important factor in determining surgical difficulty, and extensive encasement of the arteries and luminal stenosis are adverse factors that affect safe resection (13). The “dangerous triad” should be given extensive attention during surgery (14). Type IV ACMs often invade the cavernous sinus and/or encase the ICA and MCA or even invade the adventitia of the artery, making it difficult to achieve total resection; total resection was not achieved in any of these cases (Table 2).

#### 4.1.2. Relationships between tumor type and resection extent

The total resection rate of the 63 ACM cases in this study was 76.2%. The Type II ACM cases had the highest total resection rate, followed by those with Type III, but it was difficult to achieve total resection for those with Type I and IV. Only one case of a small ACM received Grade II resection among the seven cases of Type I ACMs (14.3%). If there is an intact arachnoid between Type II ACMs and the ICA, the tumor can be easily separated from the ICA (Grades I and II). In this study, 37 out of the 39 Type II cases (94.9%) achieved Simpson Grade I–II total resection (Table 2). Type III ACMs arise from the optic foramen and extend to the optic canal; the total resection rate of these ACMs is lower than that of Type II ACMs. Four of the five cases of Type III ACMs in this study received Simpson Grade I–II total resection (80%). For Type IV ACMs, the tumor base covers the entire ACP and/or invades the cavernous sinus, involving the anterior circulation and anterior visual pathway. Here, there were 12 cases of Type IV ACMs, where only six cases achieved Grade I–II total resection (50%). In Giammattei et al.'s (15) retrospective analysis of 1,208 cases of ACMs, the overall resection rate was 64.2%, with total resection of Al-Mefty Type II ACMs being the highest (92.6%), consistent with the present study's results.

#### 4.1.3. Tumor typing and surgical approach selection

Selection of Grade I approaches for ACMs essentially depends on the gross tumor volume, tumor location, and the tumor's relationship with the cavernous sinus and ICA. Selection of Grade II approaches depends on the tumor type, size, and growth. The intradural approach (Group B) has a limitation—it does not allow for exploration of the cavernous sinus; however, it is still suitable for the majority of ACMs (57.1%). Lee et al. (10) and Sade and Lee (16) provided detailed modified epidural skull base techniques (SBTs). Only 20.6% ACMs required use of the epidural approach, as shown in Table 3. The authors of this paper advocate a combination of the intradural and epidural approaches (Group C), as shown in Table 3.

The standard pterional approach for Type I ACMs can provide adequate surgical exposure and does not require conventional and complete removal of the ACP. For Grade II approaches, the intradural approach (Group B) can be selected. During surgery, if it is discovered that the tumor has invaded the optic canal or is accompanied by hyperostosis of the ACP, individualized intradural removal can be performed based on the actual situation. The modified pterional epidural approach can be selected for Type III ACMs. For Al-Mefty Type I and III ACMs, the epidural approach can be used (Group A) if the traditional intradural approach does not allow for early optic nerve identification. In Type IV ACMs, the orbitozygomatic approach is mostly used as the Level I approach. The intradural and epidural combination approach (Group C) should be selected as the Grade II approach. Mariniello et al.'s (17) study compared the traditional pterional intradural approach (32 cases) and an SBT (14 cases), which led to total tumor resection rates of 81 and 93%, respectively. The present study's individualized selection of Grade I and II approaches according to tumor type and size (Table 3) have also produced good prognoses.

### 4.2. Analysis of the factors influencing the extent of tumor resection

#### 4.2.1. Necessity and feasibility of total resection

The ideal goal for ACM is total resection. After total resection, the relapse rate is ≤ 10% during the 10-year follow-up period (6); the relapse rate of meningiomas that are not completely resected can go as high as 50%. In Czernicki et al.'s (18) report on 30 ACM cases, the postoperative relapse rate of radically resected ACMs went up to 11.8%, while cases with subtotal resection had a postoperative residual tumor progression rate of 25%. In the current study, 48 cases achieved Simpson Grade I–II total resection, with a relapse rate of 2.1% during the postoperative follow-up period. Among the 15 cases with Simpson Grade III–IV resection, there was a progression rate of up to 46.7% (7/15). Therefore, aggressive total tumor resection can prevent or delay relapses (1). The relationships between ACMs and the ICA, MCA, ACA, and nerves primarily fall under Level 0–2 relationships, and such ACMs can be completely resected. Total or radical ACM resection can reduce the risk of relapse. If total resection is impossible, the tumor volume should be minimized. However, for ACM resection, a risk and benefit assessment should be conducted to determine whether further resection will provide sufficient advantages or cause possible risks. That is, the extent of tumor resection should be correctly understood to minimize surgical risks (1).

#### 4.2.2. Factors influencing total tumor resection

① Tumor invasiveness: Invasion of the cavernous sinus and ICA is a key factor influencing the extent of tumor resection (19). Sughrue et al. (20) stated that total ACM resection involving the cavernous sinus can rarely be achieved. Risi (11) reported that total resection of only 26.7% of ACMs extending into the cavernous sinus can be achieved. In the 63 cases in the current study, nine had ACMs that invaded the cavernous sinus, and only one achieved total resection (11.1%). When an arachnoid interface exists (Level 1 or 2 relationship), even if the vessels and nerves are encased by the tumor, the vessels can still be dissected and separated from the tumor. Lack of an arachnoid interface (Level 3 relationship) indicates that there may be firm adhesion between the tumor and the vessels, and risk of vascular injury increases significantly. In the study group, seven cases had a Level 3 relationship between the tumor and anterior circulation structures, and none of them achieved total resection. Meanwhile, those with Level 0 (5 cases) and Level 1 (12 cases) relationships achieved Simpson Grade I–II total resection (100%). In conclusion, the relationship between the ACMs and anterior circulation and nerves were significantly correlated with Simpson Grade I–II total resection (*P* < 0.01), as shown in Table 2.
② Tumor size: Large tumors are more likely to invade the cavernous sinus or encase and invade the ICA and its branches, and as a result, these factors primarily prevent total ACM resection. According to the analyses conducted by Xu et al. (3), ACM size (< 3 cm) is an independent risk factor for more extensive resection. According to Goel et al. (21), the risk of surgical failure for ACM resection >3 cm is high. In the current study, there was no direct relationship between tumor size and the extent of resection (*P* = 0.14), as shown in Table 2.
③ Tumor type: The tumor type was closely related to the total resection rate (*P* < 0.001), where the total resection rate of Type I ACMs was the lowest (14.3%) and that of the Type II ACMs was the highest (94.9%; Table 2). Type IV ACMs are often treated with relatively conservative surgical strategies. Giammattei et al. (15) reported a total resection rate of 11.8% for Al-Mefty Type I ACMs, 92.6% for Type II ACMs, and 84.2% for Type III ACMs, consistent with the results in the present study. Definite information about ACM is ultimately only available intraoperatively, and correct preoperative classification is not always reliable. Therefore, it is necessary to detect whether there is tumor invasion in the optic canal during the operation, and if there is tumor invasion in the optic canal, the optic canal needs to be opened.
④ Relationship between tumors and nerves/blood vessels: The extent to which ACMs encase nerves and blood vessels is an important factor influencing the total resection rate. In the 30 cases of ACMs reported by Czernicki et al. (18), 11 cases received incomplete resection (Simpson Grade IV), as five among these had tumors with severe adhesion to cerebral arteries and six had tumors invading the cavernous sinus. In the present study, seven cases had a Level 3 relationship between the tumors and blood vessels or the anterior visual pathway, and none of them achieved total resection (Table 2). There is a significant correlation between the extent of tumor resection and the relationship between the tumors and nerves/blood vessels (*P* < 0.01).
⑤ Tumor texture and selection of level II approaches: In this study, there was a close correlation between total ACM resection and tumor texture; preoperatively determining the tumor density can help predict surgical difficulty. Here, 39 out of 41 cases of ACMs with a soft texture achieved Simpson Grade I–II total resection (95.1%, 39/41), while only nine out of 22 cases of ACMs with a hard texture achieved the same (40.9%, 9/22), showing a significant correlation between tumor texture and resection extent (*P* < 0.01; Table 2). The influence of surgical approaches on the extent of tumor resection is considered controversial. In the present study, the relationship between the Level II approaches (A, B, or C) and the extent of tumor resection showed no direct correlation (*P* = 0.936), as shown in Table 4.
⑥ ACP removal and optic canal opening: ACP removal leads to optic nerve exposure, and optic canal opening can improve optic nerve exposure and location and increase optic nerve mobility, contributing to tumor resection. The extent to which tumor resection could be done was correlated with whether ACP removal and optic canal opening were performed, as shown in Table 4.

### 4.3. Analysis of the factors influencing postoperative efficacy

#### 4.3.1. Factors influencing postoperative visual acuity

① Preoperative visual acuity level: Direct compression of the visual pathway is the most important factor leading to visual impairment in ACMs; vision problems may be reversible if decompression is performed within the ischemic penumbra window (22). Of the 60 ACM cases reported by Goel et al. (21), 55 had visual defects (91.7%) before surgery; after surgery, 14 (25%) had improved visual acuity. In cases reported by Xu et al. (3) where there was visual deterioration before surgery, 52.6% had visual acuity improvement. In the current study, the postoperative visual acuity prognosis was significantly correlated with the preoperative visual acuity level (*P* < 0.01), as shown in Table 5.
② Optic canal opening: Opening the optic canal can improve the exposure of the optic nerve and increase its mobility. In 46 ACM cases reported by Mariniello et al. (23), in the early stage, the traditional pterional approach was selected for 32 cases (Group A), of which optic canal opening was performed for only 6. In the later stage, conventional ACP removal and optic canal opening were performed for the remaining 14 cases (Group B). The results showed that postoperative visual acuity improvement was significantly higher in Group B than in Group A (80 vs. 45%). In the current study, the optic canal was opened during surgery in 22 cases, of which 21 had visual acuity improvement (95.5%, 21/22). The optic canal was not opened during surgery in 41 cases, of which 27 had visual function improvement (65.9%, 27/41). This demonstrates that intraoperative optic canal opening can improve postoperative visual acuity, as shown in Table 5.
③ Influence of ACP removal on visual function: ACP removal can increase the exposure of the surgical area, and lead to the opening of the optic canal. Pamir et al. (1) reported that ACM cases in their study observed up to 84.6% improvement in visual function when ACP was not removed. The results of the present study also showed that postoperative visual function had no correlation with whether the ACP was removed during surgery (*P* = 0.347), as shown in Table 5.
④ Influence of Grade II approaches on postoperative visual function: The influence of decompression of the optic canal and the timing of decompression on postoperative visual acuity is considered controversial. Lee et al. (10) reported 15 ACM cases where they observed that positive postoperative visual outcomes may be related to early optic nerve decompression. However, in the 43 ACM cases reported by Pamir et al. (1) and treated *via* the intradural approach, 26 cases with visual deterioration before surgery had an improvement rate of 84.6%. In the current study, postoperative visual function had no correlation with the selection of Grade II approaches (*P* = 0.794), as shown in Table 5.

#### 4.3.2. Factors influencing postoperative KPS

Russell and Benjamin (24) reported KPS improvement in 32.4% of patients and deterioration in 11.8% of patients. The average KPS 6 months after surgery (91.3 ± 9.6 points) was higher than at discharge (87.6 ± 7.8 points) and before surgery (89.3 ± 10.5 points) in the study. Tumor type was closely correlated with postoperative KPS (*P* < 0.01), while the selection of Grade II approaches and the extent of tumor resection had no correlation with postoperative KPS (*P* > 0.05), as shown in Table 6.

#### 4.3.3. Factors influencing postoperative tumor relapse

Kim et al. (7) considered GTR as an independent prognosis factor for relapse-free survival. In the 131 ACM cases reported by Xu et al. (3), 13 had tumor relapses or regrowth (11.4%), with a higher chance of relapse/regrowth in the Type III and IV ACM cases. Bassiouni et al. (6) reported tumor relapse rate of 10%, with a tumor progression rate after subtotal resection of 38%. In the present study, the overall relapse rate of the 63 cases with ACMs was observed to be 12.7%. The relapse rate in cases with Simpson Grade I–II total resection was 2.1%, and the tumor relapse/progression rate in cases with Simpson Grade III–IV resection was 46.7%. The extent of tumor resection and tumor type had a significant correlation with the postoperative relapse rate (*P* < 0.01), as shown in Table 7.

#### 4.3.4. Factors influencing postoperative complications

The tumor type and the extent of tumor invasion of blood vessels and the cavernous sinus have a significant correlation with postoperative complications. Bassiouni et al. (6) believed that the relationship between tumors and the major ICA branches or the optic nerve structure and cavernous sinus invasion are the prognostic factors influencing surgical complications and outcomes. In the 29 ACM cases reported by Wong et al. (25), the incidence of complications reached 10.3% in the perioperative period, and the incidence and mortality of permanent complications were 6.9 (visual deterioration) and 0%, respectively. In the 63 ACM cases in the current study, 4 cases (6.3%) developed permanent complications. The tumor type, the relationship between the tumors and blood vessels/nerves, cavernous sinus involvement, and the selection of Grade II approaches were the main factors influencing postoperative complications (*P* < 0.01; Table 8).

To sum up, correct classification of ACMs before surgery can aid in correctly devising surgical strategies and selecting appropriate surgical approaches, and individualized choice of ACP removal or/and optic canal opening can offer important clinical value in predicting the surgical risk and estimating the prognosis.

### 4.4. Limitations of the study

This study was a single-institution retrospective analysis, with data collected from the empirical results of a single institution, which may have led to case selection and treatment regimen bias. Furthermore, the non-random data, uneven follow-up periods, relatively short follow-up time, and relatively few cases included may not have fully reflected postoperative efficacy. The conclusions need to be verified through a prospective study with at least 5 years of follow-up data and such a study should be designed to determine the real relapse rate, which is the goal of future research.

## Data availability statement

The raw data supporting the conclusions of this article will be made available by the authors, without undue reservation.

## Ethics statement

This study was conducted with approval from the Ethics Committee of Sichuan Provincial People's Hospital. The patients/participants provided their written informed consent to participate in this study.

## Author contributions

Conception and design of the research: L-HC, H-ZH, and FW. Acquisition of data: KS, H-ZH, R-XX, and YX. Analysis and interpretation of the data: L-HC, YX, H-ZH, and FW. Statistical analysis: KS, L-HC, and FW. Obtaining financing, writing of the manuscript, and critical revision of the manuscript for intellectual content: L-HC and R-XX. All authors read and approved the final draft.
